# Sir Humphry Davy’s Choice

**Published:** 1879-10

**Authors:** 


### SIR HUMPHREY DAVY'S CHOICE.

The subjoined beautiful thoughts are from Sir Humphrey Davy's "*Salmonia*":

"I envy no quality of mind or intellect in others, be it genius, power, wit, or fancy; but if I could choose what would be most delightful, and I believe would be most useful to me, I should prefer a firm religious belief to every other blessing: for it makes life a discipline of goodness; creates new hopes when all other hopes vanish; and throws over the decay, the destruction of existence, the most gorgeous of all lights; awakens life even in death, and from corruption and decay calls up beauty and divinity; makes an instrument of ill-fortune and shame the ladder of ascent to Paradise; and far above all combinations of earthly hopes, calls up the most delightful visions of palms and amaranths, the Gardens of the Blest, the security of everlasting joys, where the sensualist and the skeptic view only gloom, decay, annihilation. and despair."

